# Microbial Cell Factory for Efficiently Synthesizing Plant Natural Products via Optimizing the Location and Adaptation of Pathway on Genome Scale

**DOI:** 10.3389/fbioe.2020.00969

**Published:** 2020-08-14

**Authors:** Bo Yang, Xudong Feng, Chun Li

**Affiliations:** ^1^SynBio Research Platform, Collaborative Innovation Center of Chemical Science and Engineering, Key Laboratory of Systems Bioengineering, Ministry of Education, School of Chemical Engineering and Technology, Tianjin University, Tianjin, China; ^2^Institute for Synthetic Biosystem/Department of Biochemical Engineering, School of Chemistry and Chemical Engineering, Beijing Institute of Technology, Beijing, China; ^3^Key Laboratory for Industrial Biocatalysis, Ministry of Education, Department of Chemical Engineering, Tsinghua University, Beijing, China

**Keywords:** plant natural products, microbial cell factory, pathway location, adaptation, heterologous pathway

## Abstract

Plant natural products (PNPs) possess important pharmacological activities and are widely used in cosmetics, health care products, and as food additives. Currently, most PNPs are mainly extracted from cultivated plants, and the yield is limited by the long growth cycle, climate change and complex processing steps, which makes the process unsustainable. However, the complex structure of PNPs significantly reduces the efficiency of chemical synthesis. With the development of metabolic engineering and synthetic biology, heterologous biosynthesis of PNPs in microbial cell factories offers an attractive alternative. Based on the in-depth mining and analysis of genome and transcriptome data, the biosynthetic pathways of a number of natural products have been successfully elucidated, which lays the crucial foundation for heterologous production. However, there are several problems in the microbial synthesis of PNPs, including toxicity of intermediates, low enzyme activity, multiple auxotrophic dependence, and uncontrollable metabolic network. Although various metabolic engineering strategies have been developed to solve these problems, optimizing the location and adaptation of pathways on the whole-genome scale is an important strategy in microorganisms. From this perspective, this review introduces the application of CRISPR/Cas9 in editing PNPs biosynthesis pathways in model microorganisms, the influences of pathway location, and the approaches for optimizing the adaptation between metabolic pathways and chassis hosts for facilitating PNPs biosynthesis.

## Introduction

Plant natural products (PNPs) are secondary metabolites that are mainly used for defense and signal transduction in plants (Marienhagen and Bott, 2012). They have complex structures and various physiological as well as pharmacological activities. For instance, glycyrrhizin, betulinic acid, paclitaxel and resveratrol have antitumor activities, while lycopene, β-carotene, and astaxanthin possess antioxidant properties (Lü et al., 2016; Zhao et al., 2017; Zhang L. et al., 2019; Chen et al., 2020; Sun et al., 2020). Obtaining natural products through extraction from cultivated plants faces the problem of low natural content, uncertain climate factors and destruction of the ecological environment (Zhao and Li, 2018; Sun et al., 2019). With the rapid advances of synthetic biology, the production of PNPs in microbial cell factories has paved the way for large-scale industrial production by shortening the synthesis cycle and reducing the difficulty of product separation (Liu et al., 2017).

There three main genome editing tools for pathway modification and regulation *in vivo*, including zinc-finger nucleases (ZFNs), transcription activation-like effector nucleases (TALENs), and the clustered regularly interspaced short palindromic repeats (CRISPR) system. In microorganisms, CRISPR can accurately edit and regulate the metabolic pathways of PNPs in an efficient manner without selection markers (Hou et al., 2018). However, the introduction of heterologous pathways usually disrupts the intracellular metabolic balance (Liu et al., 2019), while the integration loci of heterologous expression cassettes affect enzyme expression and product accumulation (Englaender et al., 2017). Thus, appropriately chosen loci can enhance the stable expression of heterologous genes. Additionally, organelles with a high local concentration of the substrates are a suitable location for PNPs synthesis. Numerous studies showed that the balance between endogenous and exogenous pathways contributed to the efficient synthesis of products (Zhang et al., 2015; Park et al., 2018), and the highest expression level of enzymes did not necessarily maximize the yield (Kang et al., 2018). At the same time, the adaptation of the chassis cells to the heterologous pathway is equally important. Traditional metabolic engineering is employed to regulate potential pathways and restore intracellular metabolic balance, which relies on targets obtained by analyzing metabolic flux distribution, reaction mechanisms or metabolic network models. However, a finite number of target modifications cannot produce the full possible variety of genotypes and phenotypes to select the optimal phenotypes. Reprogramming the expression of multiple genes is a complementary strategy, relying on approaches such as multiplex automated genome engineering (MAGE; Wang et al., 2009), or synthetic chromosome rearrangement and modification by loxP-mediated evolution (SCRaMbLE; Standage-Beier and Wang, 2017), which are tools for rewriting genomes. Moreover, heterologous synthesis of PNPs often involves multi-step enzymatic reactions, and synthetic scaffolds can improve the catalytic activity of the system to some extent (Zhang, 2011). To address the demand for multiple auxotrophic markers, the controllable decentralized assembly strategy was established and recyclable markers were employed for iterative integration (Xie et al., 2014). This review covers recent studies on pathway location and strategies for optimizing the fitness and intracellular metabolism of the chassis cells to promote the production of PNPs.

## Application of CRISPR/Cas9 in Pathway Integration and Regulation

In 2013, the gene editing function of the CRISPR/Cas9 system was first verified in mammals (Cong et al., 2013). Soon afterward, CRISPR/Cas9 was universally applied in microorganisms. The CRISPR-mediated multi-locus gene integration strategy was developed to efficiently edit the β-carotene synthesis pathway in *Saccharomyces cerevisiae* (Ronda et al., 2015). Another significant research was that GTR-CPISPR could edit eight genes simultaneously with an efficiency up to 84% (Zhang Y. et al., 2019). The Cas9-based toolkit with high efficiency for integrating cassettes increased taxadiene production by 25-fold (Apel et al., 2017). In addition to genome editing functions, some derivatives have been exploited for precise and rapid regulation of gene expression. CRISPR interference (CRISPRi) can be used to regulate the transcription of multiple sites in the genome by fusing activators or repressors with catalytically-inactive Cas9 (dCas9) protein (Gilbert et al., 2013). Harnessing CRISPRi to synchronously down-regulate seven genes in the competing pathway boosted β-amyrin biosynthesis (Ni et al., 2019). The production of other PNPs such as pinosylvin (Wu et al., 2017a), resveratrol (Wu et al., 2017b), O-methylated anthocyanin (Cress et al., 2017), and α-amyrin (Yu et al., 2018) in microbes was also successfully enhanced using CRISPRi. Zalatan et al. (2015) extended sgRNA with modular RNA domains to form the scaffold RNA (scRNA), which can not only recognize target sequences specifically but also recruit RNA-binding proteins fused with effectors. The expression of multiple scRNAs made up for the defect of CRISPRi and achieved diverse types of regulation at respective loci in parallel. Employing the scRNA system, the best gRNA screened from 101 candidates was chosen to regulate multiple genes in the carotenoid biosynthesis pathway (Jensen et al., 2017). It is worth noting that disparate gRNAs competing for the same Cas9 is a likely limiting factor in scRNA systems. CRISPR-AID, an orthogonal tri-functional system, enabled the concurrent upregulation, downregulation and deletion of genes in *S. cerevisiae*, achieving the modular regulation of metabolic networks (Lian et al., 2017). Successful examples of the application of CRISPR/Cas9 and its derivatives in PNPs biosynthesis are summarized in Table 1.

**TABLE 1:** The application of CRISPR/Cas9 and derived technologies in the biosynthesis of PNPs.

Species	Products	Method	Regulation strategy	Culture method	Titer	References
*E. coli*	β-carotene	CRISPR/Cas9	Genomic modification	Fed-batch (5 L)	2.0 g/L	Li et al., 2015
*S. cerevisiae*	Mevalonate	CRISPR/Cas9	Multi-gene disruption	–	1.5 mg/L (41-fold)	Jakounas et al., 2015
*Yarrowia lipolytica*	Lycopene	CRISPR/Cas9	Multi-gene integration	Shake flask (250 mL)	8.6-fold	Schwartz et al., 2017
*S. cerevisiae*	Valencene	CRISPR/Cas9	Knock-out or down-regulation	Fed-batch (3 L)	539.3 mg/L (160-fold)	Chen et al., 2019
*S. cerevisiae*	guaia-6,10 (14)-diene	CRISPR/Cas9	Gene integration	Fed-batch (5 L)	0.8 g/L	Siemon et al., 2020
*Candida tropicalis*	β-carotene	CRISPR/Cas9	Multi-gene deletion or mutation	–	0.23 mg/g DCW	Zhang L. et al., 2020
*Yarrowia lipolytica*	β-carotene	CRISPR/Cas9	Gene integration	Fed-batch (5 L)	4.5 g/L	Zhang X.K. et al., 2020
*S. cerevisiae*	β-carotene	CrEdit	Multi-loci gene integration	–	12.7 mg/L	Ronda et al., 2015
*S. cerevisiae*	Patchoulol	Cas-3P	Multiplexed and sequential editing	Shake flask (250 mL)	20 mg/L	Li et al., 2020
*S. cerevisiae*	Taxadiene	Cas9-based toolkit	Including Cas9-sgRNA plasmids, promoters, protein tags	Test tube (5 mL)	20 mg/L (25-fold)	Apel et al., 2017
*E. coli*	Naringenin	CRISPRi	Multi-gene repression	Shake flask	421.6 mg/L (7.4-fold)	Wu et al., 2015
*E. coli*	Lycopene (-)-α-bisabolol	CRISPRi	Regulatable repression	Shake flask	50.6 mg/L 8.8-fold	Kim et al., 2016
*E. coli*	Peonidin 3-O-glucoside	CRISPRi	Silence expression	Shake flask (125 mL)	56 mg/L (2-fold)	Cress et al., 2017
*E. coli*	Pinosylvin	CRISPRi	Multi-gene repression	Shake flask (500 mL)	281 mg/L	Wu et al., 2017a
*E. coli*	Resveratrol	CRISPRi	Multi-gene down-regulation	Shake flask	304.5 mg/L	Wu et al., 2017b
*S. cerevisiae*	α-amyrin	CRISPRi	Down-regulation	–	11.97 mg/L	Yu et al., 2018
*S. cerevisiae*	β-amyrin	CRISPRi	Multi-gene one-step down-regulation	Fed-batch (2.5 L)	156.7 mg/L(1.44-fold)	Ni et al., 2019
*S. cerevisiae*	β-carotene	CRISPR-AID	Synchronous up/down-regulation, deletion	–	3-fold	Lian et al., 2017
*Ogataea polymorpha*	Resveratrol	CMGE	Multi-gene knock-out, integration	Shake flask (500 mL)	97.23 mg/L (20.73-fold)	Wang L. et al., 2018

## Optimization of the Pathway Location

Microbial cell factories usually have to efficiently express multiple heterologous genes, and the introduction of episomal plasmids can impose a heavy metabolic burden. At the same time, it was found that the genetic stability of the host decreases when multiple plasmids are used in the same cell (Yan et al., 2005). By contrast, chromosomal integration of target genes is more stable and does not require a selective pressure (Zhu et al., 2018). For example, integrating target genes into the native GAL1 locus of engineered *S. cerevisiae* increased the oleanolic acid titer by 3.6 times compared with the strain utilizing multiple plasmids (Zhao et al., 2018). Optimizing the chromosomal integration sites of metabolic pathways and compartmentalizing metabolic pathways in organelles are both viable strategies for improving the synthesis of PNPs.

### Chromosomal Integration Loci of Metabolic Pathways

The integration loci of heterologous genes in the genome influence enzyme expression levels in various hosts, such as *Escherichia coli* (Englaender et al., 2017), yeast (Guo et al., 2018), actinomycetes (Bilyk et al., 2017), and *Lactococcus lactis* (Thompson and Gasson, 2001), and may even affect the titer of products (Bilyk et al., 2017). Enzymes with easily detectable activities or fluorescent proteins are normally chosen to characterize the expression intensity of sites, providing important guidance for the construction of efficient microbial cell factories.

In one study, a total of 1044 loci within the whole genome of *S. cerevisiae* were analyzed and the largest difference in expression levels among them was 13 times (Wu X.-L. et al., 2017). Sites with low expression intensity were mainly located near telomeres and centromeres (Ottaviani et al., 2008), and the loci with high expression levels were adjacent to autonomously replicating sequences. The robustness of position effect was even stronger with different promoters (Bai Flagfeldt et al., 2009), reporter genes, and carbon sources (Wu X.-L. et al., 2017). After taking into account the impact of chromosomal location on gene expression and growth rate, 11 out of 14 genomic loci in *S. cerevisiae* were found to be suitable for pathway integration (Mikkelsen et al., 2012). The introduction of eight genes at four sites enabled high indolylglucosinolate production. In *Yarrowia lipolytica*, some loci that could enhance the stable expression of the β-carotene (Zhang X.K. et al., 2020) or lycopene (Schwartz et al., 2017) biosynthesis pathways were screened out. Similar to plasmid expression, the copy number of the integration site also has a considerable effect on product synthesis (Wang L. et al., 2018). A fused expression cassette containing three genes for resveratrol biosynthesis was inserted into the multi-copy rDNA cluster in *Ogataea polymorpha* and strains with copy numbers ranging from 1 to 10 were obtained. Within this range, the increase of copy numbers of the expression cassette increased the resveratrol titer 20 times in comparison to integrating the three genes into scattered single-copy loci, respectively.

However, the protein expression level of the biosynthetic enzymes was not always positively correlated with the yield of PNPs (Englaender et al., 2017). Integration loci in the genome affect the efficiency of pathways, and inappropriate selection of sites may even hinder product synthesis. The effects of integration sites on strain growth, genetic stability, and product synthesis should be taken into account comprehensively.

### Subcellular Compartmentalization of Metabolic Pathways

Subcellular organelles, such as mitochondria, peroxisomes, and endoplasmic reticulum, have complex structures and act as independent membrane-bound compartments, which leads to higher local concentrations of substrates and generates physical separation between products and competing pathways (Huttanus and Feng, 2017). Compartmentalizing metabolic pathways in organelles achieved by fusing proteins with targeting signal tags can enhance the reaction rate and product synthesis efficiency.

Farnesyl diphosphate, which is an important intermediate in carotenoid production, is abundant in peroxisomes (Kovacs et al., 2002). When heterologous enzymes of the lycopene synthesis pathway were targeted into peroxisomes, it remarkably boosted the titer of lycopene to 73.9 mg/L in *Komagataella phaffii* (Bhataya et al., 2009). Another organelle commonly used in compartmentalization studies is mitochondria. Employing mitochondrion-targeted enzymes resulted in 8 and 20-fold increase in valencene and amorphadiene production, respectively (Farhi et al., 2011). The concentration of acetyl-CoA in mitochondria is 20–30 times higher than that in the cytoplasm (Galdieri et al., 2014). Many studies focused on the mitochondrial acetyl-CoA pool to promote the production of PNPs such as amorpha-4,11-diene (Yuan and Ching, 2016), geraniol, 8-hydroxygeraniol, and nepetalactol (Yee et al., 2019). Recently, dual engineering of metabolic pathways in the cytoplasm and organelles was performed for high-level PNPs production. Assembling the complete MVA pathway in peroxisomes or mitochondria, together with the cytoplasmic pathway, boosted the output of α-humulene (Zhang C et al., 2020), and linalool (Zhang Y et al., 2020), respectively.

## Adaptation of Chassis Cells to Heterologous Pathways

**FIGURE 1:**
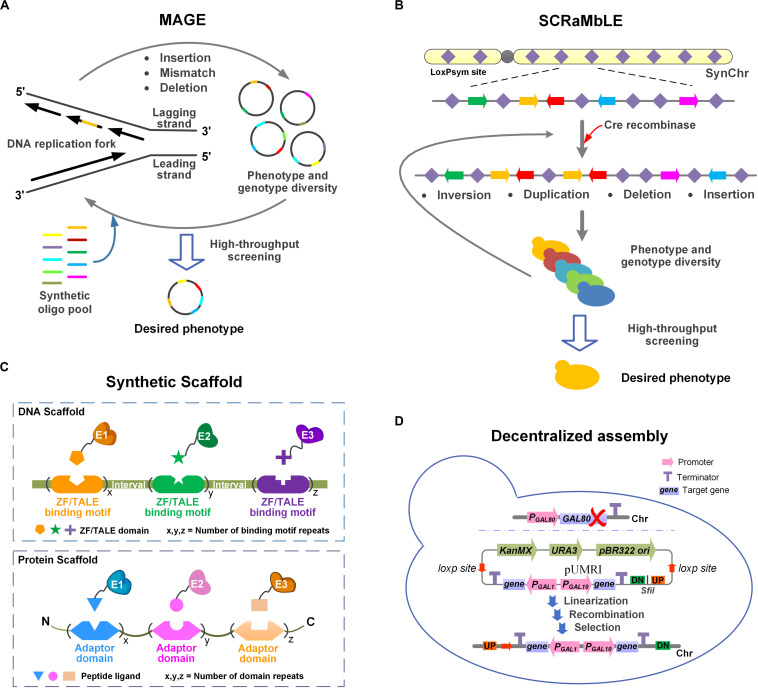
Schematic illustration of four strategies for adapting the chassis cells to the heterologous pathway. **(A)** MAGE. Insertions, mismatches or deletions can be introduced at multiple specific sites in the genome by MAGE. Multiple iterations result in phenotypic and genotypic diversity. **(B)** SCRaMbLE. Genomic rearrangements (inversions, duplications, deletions, and insertions) occur at any two loxPsym sites stochastically. Phenotypic and genotypic diversity was obtained through iterative cycling. **(C)** Synthetic scaffold. In DNA scaffold, the target enzymes are fused with ZF/TALE domains through flexible linkers. In protein scaffold, the target enzymes are fused with peptide ligands via flexible linkers. **(D)** Decentralized assembly. GAL80 was deleted to obtain the GAL regulatory system. pUMRI, a marker-recyclable integrative toolbox with double selection was used to integrated genes into scattered loci. The controllable system and pUMRI constitute the decentralized assembly strategy.

The introduction of a heterologous pathway almost always negatively affects metabolic homeostasis. It is therefore necessary to improve the fitness on a genome-wide level for efficient PNPs production. Here, we discuss the four strategies MAGE, SCRaMbLE, synthetic scaffolds, and decentralized assembly (Figure 1), which can be used to adapt the chassis to the introduced pathway.

### MAGE

In the phage λ-Red recombination system, single-stranded DNA (ssDNA) binding protein β promotes homologous complementarity between ssDNA and the lagging strand of the replication fork during DNA replication. Consequently, sequences are integrated into the genome of offspring cells, leading to allelic replacement (Ellis et al., 2001). Based on ssDNA recombination, MAGE was developed to precisely and simultaneously program multiple specific sites (insertions, mismatches, or deletions) on a genome-wide scale (Wang et al., 2009). A total of 24 endogenous genes in the metabolic pathway were modified using a synthetic oligos library, which optimized the production of lycopene in *E. coli* (Wang et al., 2009). Among them, degenerate RBS sequences that differentially regulate gene expression and nonsense mutations were inserted for inactivation. After 3 days of evolution, mutants with a 5-fold increase in yield were obtained.

However, the inefficiency of inserting large fragments by MAGE limited the modification of genetic elements. Applying switchable co-selection markers, multiple T7 promoters were introduced into 12 genomic loci simultaneously, and a combination that maximized indigo synthesis was obtained (Wang et al., 2012). Large combinatorial populations were generated in each round of MAGE, including unproductive cheaters. To ease the screening burden, toggled selection was devised to remove cheaters (Raman et al., 2014). Oligos were targeted to SD sequences of candidate genes and toggled selection after each round of evolution resulted in naringenin production of up to 61 mg/L. In some cases, the biosynthesis of PNPs depended on NADPH, and insufficient NADPH restricted PNPs production. An RBS mutant library of four genes in the Entner-Doudoroff pathway was constructed and 40 cycles of MAGE generated mutants with a high NADPH pool, which led to a 97% increase in the titer of neurosporene (Ng et al., 2015). A common feature of these studies was the use of *mutS*-deficient *E. coli*, which could improve recombination efficiency. Strain-independent MAGE was developed to reduce the accumulation of undesired mutations and to broaden the range of applicable hosts (Ryu et al., 2014). Ryu et al. integrated a suicide plasmid carrying the λ Red recombination system into the *mutS* locus to switch MutS activity in *E. coli*. By constructing a mutant library of 5′-untranslational region of genes, MAGE fine-tuned the expression level of enzymes and resulted in a 38.2-fold improvement of the curcumin titer (Kang et al., 2018).

Due to the complicated genetic regulatory system in eukaryotes, the effect of employing MAGE directly is not ideal (Si et al., 2017). Researchers developed an oligo-mediated recombination method in *S. cerevisiae*, referred to as yeast oligo-mediated genome engineering (YOGE; DiCarlo et al., 2013). After inactivating the mismatch-repair system, overexpressing DNA recombinase and optimizing the reaction conditions, the editing efficiency was increased to 1%, but it was still low. With the in-depth investigation of the mechanism, eukaryotic MAGE (eMAGE) was established for multiplex genome engineering (Barbieri et al., 2017). The efficiency of precisely editing a single nucleotide exceeded 40%, and no additional mutations were introduced at the target sites. Apart from slowing DNA replication, the complementarity of ssDNA oligodeoxynucleotides (ssODNs) with the lagging strand rather than leading strand was equally conducive to allelic replacement. Incorporation of ssODNs at the replication fork formed combinatorial genomic diversity and facilitated β-carotene production in *S. cerevisiae* (Barbieri et al., 2017).

To address the tediousness of oligonucleotide design, two web-based tools, MODEST (Bonde et al., 2014), and Merlin (Quintin et al., 2016), were developed. Iterative incorporation of ssDNA or ssODNs promoted sequence diversity. MAGE and derived technologies enable the introduction of genomic diversity, making it easier to evolve the desired phenotype compared with conventional methods and forming a large mutant library. This technology has been successfully applied to prokaryotes and eukaryotes to balance biosynthesis and cell growth, and improve the adaptation of hosts to heterologous pathways. However, the prerequisite for utilizing MAGE is to identify target genes that affect product synthesis and to possess suitable high-throughput screening methods, which has limited its wider application.

### SCRaMbLE

In the international Synthetic Yeast Genome Project (Sc2.0), bidirectional loxPsym sites were inserted downstream of each non-essential gene (Kannan and Gibson, 2017). With Cre recombinase induction, rearrangements (deletion, insertion, duplication, and inversion events) occurred at any two loxPsym sites stochastically, resulting in the SCRaMbLE technique (Jin et al., 2018). The artificial rearrangement of synthetic chromosomes on the genome-wide scale accelerates strain evolution to acquire the expected phenotypes (Wang J. et al., 2018). The genotypes were further analyzed by PCRTag and whole genome sequencing to identify the structural variations, providing an important reference for deciphering mechanism (Ma et al., 2019).

When the synthetic yeast strains containing a Cre recombinase plasmid were cultured, even without adding estradiol, a fraction of strains would switch on SCRaMbLE and their growth was slightly affected (Annaluru et al., 2014), indicating leaky expression of the plasmid. To resolve this, a genetic AND gate switch was devised to precisely control SCRaMbLE (Jia et al., 2018). A galactose-inducible promoter was used to regulate the expression of a fusion protein composed of Cre and an estrogen-binding domain. Therefore, only when galactose and estradiol were present simultaneously, genomic rearrangement can be triggered. Adopting an AND gate switch in synV haploid yeast raised the production of carotenoids by 50%. Heterozygous diploid yeasts with synthetic chromosomes and wild type counterparts were more likely to generate larger structural variations than haploid (Li et al., 2019). The synIII&V diploids formed by mating increased the likelihood of genome diversification and exhibited a 6.29- to 7.81-fold enhancement of the carotenoid yield (Jia et al., 2018). A large number of beneficial rearrangements was accumulated through multiplex SCRaMbLE iterative cycling, promoting the carotenoid synthesis up to 38.8 times.

In addition to the issue of leaky expression from plasmids, it is necessary to establish high-throughput screening methods to broaden the application of SCRaMbLE. In order for rapidly screening mutants after SCRaMbLE rearrangement, an ultra-fast LC-MS method using a guard column to substitute a standard analytical column was employed to boost betulinic acid production in *S. cerevisiae*, reducing the detection time per sample from 5 min to 84 s (Gowers et al., 2020). Ultimately, multiplex nanopore sequencing was utilized to identify the rearrangements in the high-yield strains and establish genotype-phenotype correlation.

To settle the problem that the exogenous synthesis pathway and the chassis cannot be optimized synchronously (Keasling, 2012), SCRaMbLE-in composed of an *in vitro* recombinase kit and *in vivo* chromosome rearrangement system was constructed to promote β-carotene biosynthesis (Liu et al., 2018). The recombinase toolkit inserted regulatory elements upstream of candidate genes to achieve different expression intensities. Apart from Cre, the recombinases Dre and VCre were used *in vitro* to construct the library. The *in vivo* rearrangement system induced by Cre randomly integrated the metabolic pathway into the engineered genome, leading to massive rearrangements of chromosomes. The β-carotene yield was increased by 2-fold through genomic rearrangement in comparison to integrating the pathway into the HO locus. The bottleneck of product biosynthesis can be solved through SCRaMbLE. In the process of astaxanthin production, the combination of *crtZ* and *crtW* from different sources was optimized stochastically. Among the darker-red colonies generated by SCRaMbLE, the highest yield of astaxanthin was boosted to 8.51 times (Qi et al., 2020).

Instead of using the inducer estradiol, which was toxic to humans, light-controlled SCRaMbLE (L-SCRaMbLE) was developed (Hochrein et al., 2018). The N-terminal and C-terminal of split Cre fused with chromophore-binding photoreceptor phytochrome B (PhyB) and phytochrome interacting factor PIF3, respectively, to constitute L-SCRaMbLE. Upon red light illumination, split Cre recombined and induced random rearrangements of genes among loxPsym sites. Compared with estradiol-inducible system, L-SCRaMbLE had lower recombination efficiency and mediated the recombination in a phycocyanobilin- and light-dependent manner.

Due to insufficient understanding of stress response mechanisms in microorganisms, some genes related to product synthesis were omitted during modification, which may sometimes be remedied via SCRaMbLE. This black-box approach provides a platform for rapid generation of phenotypic and genotypic diversity. Multiple rounds of SCRaMbLE were found to improve the production further (Liu et al., 2018). Meanwhile, an enormous mutant library was formed, and high-throughput screening method was a prerequisite for its wide application. Analyzing the differences in the expression of genes adjacent to rearrangement loci and the perturbation of the global metabolic network by the rearrangements is conductive to identifying possible regulatory mechanisms, which provides important reference for further rational modification to maximize PNPs synthesis in microbes.

### Synthetic Scaffold

Some enzymes of heterologous pathways are located in different positions intracellularly. For multienzyme reaction systems, the large distance among different enzymes or between enzymes and substrates would restrict product synthesis (Morgado et al., 2018). The substrate channel formed by enzymes assembled on a scaffold shortens the distance between the active sites of different enzymes and alleviates the inhibitory effects of toxic intermediates (Horn and Sticht, 2015). DNA and protein scaffold have been adopted to facilitate PNPs synthesis in cell factories. The expression levels and unit numbers of scaffolds, the stoichiometric ratio and spatial orientation of enzymes, and the interval among enzymes are important parameters for scaffold optimization. To prevent the close proximity of multiple enzyme complexes from blocking access to the substrate binding site, flexible linkers are used in the scaffold. RNA scaffold has also been investigated, and was devised as discrete, one-dimensional, two-dimensional or triangular structure to promote the output of hydrogen (Delebecque et al., 2011), pentadecane and succinate (Sachdeva et al., 2014). Nonetheless, RNA scaffold is rarely used in the biosynthesis of PNPs. A brief comparison of three synthetic scaffolds was made in Supplementary Table S1.

#### DNA Scaffold

Zinc fingers (ZFs) can bind to specific DNA sequences and form a multienzyme complex with DNA scaffold (Greisman and Pabo, 1997). Fusing two key enzymes with Zif268 and PBSII ZF domains led to a nearly 5-fold increase in resveratrol production (Conrado et al., 2012). Similarly, the assembly of three enzymes in the lycopene synthesis pathway increased the production up to 4.7 times in *E. coli* (Xu et al., 2020). Apart from ZFs, transcription activator-like effectors (TALEs) are also commonly used to construct DNA scaffold (Moscou and Bogdanove, 2009). The system composed of TALEs and corresponding DNA scaffold considerably increased the titer of indole-3-acetic acid by closely linking the two enzymes in the pathway (Zhu et al., 2016). The plasmid copy number of DNA scaffold was also found to affect the co-localization of heterologous enzymes (Xie et al., 2019).

#### Protein Scaffold

Adaptor domains SH3, SH2, PDZ, and GTPase binding domain (GBD) have strong affinity with peptide ligands and are widely used in protein scaffold (Horn and Sticht, 2015). In *E. coli*, the production of mevalonate was enhanced 77-fold by anchoring key enzymes fused with peptide ligands to the corresponding aptamer domain (Dueber et al., 2009). Based on this study, Zhao et al. (2015) adopted nine synthetic protein scaffolds from Dueber’s research to capture and assemble enzymes for catechin biosynthesis in *E. coli*. They found that genes from diverse sources could also cause significant differences in scaffold function. With similar protein domains, the optimal scaffold (GBD_1_SH3_2_PDZ_4_) increased the yield of resveratrol 2.7 times, compared with using the fusion protein in *S. cerevisiae* (Wang and Yu, 2012). Recently, SpyCatcher/SpyTag, and SnoopCatcher/SnoopTag were selected as protein tags to covalently conjugate enzymes in the mevalonate pathway, enhancing the titers of lycopene and astaxanthin to varying degrees (Qu et al., 2019).

### Decentralized Assembly

Constructing controllable and genetically stable heterologous multi-gene metabolic pathways is an effective strategy to promote product biosynthesis. In order to avoid homologous recombination among multiple repeated sequences at the same location (Blount et al., 2012), genes were integrated into different loci on the chromosome for stable expression. To achieve this, a decentralized assembly strategy comprising integrative plasmids (pMRI) with recyclable markers and GAL regulatory system was developed (Xie et al., 2014). The pMRI plasmids contain ready to use *loxP-KanMX-pBR322ori-loxP* as the selection marker, which could be recombined and removed under Cre induction to realize the recyclable utilization of the marker. All candidate genes involved in β-carotene biosynthesis were controlled by *GAL1-GAL10* bidirectional promoters. The knockout of GAL80 enabled high-glucose inhibition and low-glucose induction, so that the glucose concentration could regulate the switching time of the integrated pathway. The dynamic regulation of the assembled pathway balanced the metabolic flux between regular cellular activities and product accumulation, shifting more intracellular resources to β-carotene synthesis.

To simplify marker excision and shorten the integration period, pUMRI with the marker *loxp-kanMX-URA-loxp* was designed to substitute pMRI (Lv et al., 2016). The marker was removed through low-frequency mitotic recombination and colony counterselection. The improved decentralized assembly strategy was applied to the synthesis of isoprenoids (Ye et al., 2017) and lycopene (Zhou et al., 2018).

## Conclusion and Perspective

The location of metabolic pathways and the adaptation of the chassis cells to the heterologous pathways have a significant influence on PNPs biosynthesis. However, there are relatively few studies on the effects of different integration loci on PNPs production, which should be explored in the future. When targeting metabolic pathways to organelles, the possible negative effects of targeting signal tags on enzyme activity need to be considered. Although the genomic diversity generated by whole-genome engineering methods such as MAGE or SCRaMbLE can greatly accelerate strain evolution, high-throughput screening methods are required to identify beneficial mutants in the enormous mutant library, which is a pivotal step during laboratory evolution. There are few high-throughput screening methods. In most cases, these methods are only applicable to the specific reactions. The lack of efficient screening methods is a universal bottleneck in current research. For genome editing, an automated high-throughput screening platform is an important direction for future research. In addition to cytoplasmic protein scaffold, emerging membrane-bound scaffold is expected to improve PNPs biosynthesis. Examples include cohesion-based protein scaffold located on the membranes of lipid droplets (Lin et al., 2017), Tat-assisted scaffold in the thylakoid membrane (Henriques de Jesus et al., 2017), and membrane steroid-binding protein-mediated scaffold anchored to the ER membrane (Gou et al., 2018). To avert chromosomal rearrangement between repeated loxP segments, seamless recombination can be performed to optimize the decentralized assembly strategy. As synthetic biology tools and strategies move forward, these challenges will be addressed gradually. Microbial cell factories have the potential to achieve efficient synthesis and large-scale industrial production of PNPs.

## Author Contributions

All authors contributed to conception and design of the study. BY participated in searching and analyzing literature for this review and wrote the manuscript. XF and CL edited and corrected the manuscript. All authors approved the submitted version.

## Conflict of Interest

The authors declare that the research was conducted in the absence of any commercial or financial relationships that could be construed as a potential conflict of interest.

## References

[B1] Annaluru N. Muller H. Mitchell L. A. Ramalingam S. Stracquadanio G. Richardson S. M. (2014). Total synthesis of a functional designer eukaryotic chromosome. *Science* 344 55–58. 10.1126/science.1249252 24674868 PMC4033833

[B2] Apel A. R. d’Espaux L. Wehrs M. Sachs D. Li R. A. Tong G. J. (2017). A Cas9-based toolkit to program gene expression in *Saccharomyces cerevisiae*. *Nucleic Acids Res.* 45 496–508. 10.1093/nar/gkw1023 27899650 PMC5224472

[B3] Bai Flagfeldt D. Siewers V. Huang L. Nielsen J. (2009). Characterization of chromosomal integration sites for heterologous gene expression in *Saccharomyces cerevisiae*. *Yeast* 26 545–551. 10.1002/yea.1705 19681174

[B4] Barbieri E. M. Muir P. Akhuetie-Oni B. O. Yellman C. M. Isaacs F. J. (2017). Precise editing at DNA replication forks enables multiplex genome engineering in Eukaryotes. *Cell* 171 1453–1467. 10.1016/j.cell.2017.10.034 29153834 PMC5995112

[B5] Bhataya A. Schmidt-Dannert C. Lee P. C. (2009). Metabolic engineering of *Pichia pastoris* X-33 for lycopene production. *Process Biochem.* 44 1095–1102. 10.1016/j.procbio.2009.05.012

[B6] Bilyk B. Horbal L. Luzhetskyy A. (2017). Chromosomal position effect influences the heterologous expression of genes and biosynthetic gene clusters in *Streptomyces albus* J1074. *Microb. Cell Fact.* 16:5. 10.1186/s12934-016-0619-z 28052753 PMC5209838

[B7] Blount B. A. Weenink T. Ellis T. (2012). Construction of synthetic regulatory networks in yeast. *FEBS Lett.* 586 2112–2121. 10.1016/j.febslet.2012.01.053 22309848

[B8] Bonde M. T. Klausen M. S. Anderson M. V. Wallin A. I. N. Wang H. H. Sommer M. O. A. (2014). MODEST: a web-based design tool for oligonucleotide-mediated genome engineering and recombineering. *Nucleic Acids Res.* 42 W408–W415. 10.1093/nar/gku428 24838561 PMC4086063

[B9] Chen H. Zhu C. Zhu M. Xiong J. Ma H. Zhuo M. (2019). High production of valencene in *Saccharomyces cerevisiae* through metabolic engineering. *Microb. Cell Fact.* 18:195. 10.1186/s12934-019-1246-2 31699116 PMC6839068

[B10] Chen R. Yang S. Zhang L. Zhou Y. J. (2020). Advanced strategies for production of natural products in yeast. *iScience* 23:100879. 10.1016/j.isci.2020.100879 32087574 PMC7033514

[B11] Cong L. Ran F. A. Cox D. Lin S. Barretto R. Habib N. (2013). Multiplex genome engineering using CRISPR/Cas systems. *Science* 339 819–823. 10.1126/science.1231143 23287718 PMC3795411

[B12] Conrado R. J. Wu G. C. Boock J. T. Xu H. Chen S. Y. Lebar T. (2012). DNA-guided assembly of biosynthetic pathways promotes improved catalytic efficiency. *Nucleic Acids Res.* 40 1879–1889. 10.1093/nar/gkr888 22021385 PMC3287197

[B13] Cress B. F. Leitz Q. D. Kim D. C. Amore T. D. Suzuki J. Y. Linhardt R. J. (2017). CRISPRi-mediated metabolic engineering of *E. coli* for O-methylated anthocyanin production. *Microb. Cell Fact.* 16:10. 10.1186/s12934-016-0623-3 28095853 PMC5240198

[B14] Delebecque C. J. Lindner A. B. Silver P. A. Aldaye F. A. (2011). Organization of intracellular reactions with rationally designed RNA assemblies. *Science* 333 470–474. 10.1126/science.1206938 21700839

[B15] DiCarlo J. E. Conley A. J. Penttilä M. Jäntti J. Wang H. H. Church G. M. (2013). Yeast Oligo-Mediated Genome Engineering (YOGE). *ACS Synth. Biol.* 2 741–749. 10.1021/sb400117c 24160921 PMC4048964

[B16] Dueber J. E. Wu G. C. Malmirchegini G. R. Moon T. S. Petzold C. J. Ullal A. V. (2009). Synthetic protein scaffolds provide modular control over metabolic flux. *Nat. Biotechnol.* 27 753–759. 10.1038/nbt.1557 19648908

[B17] Ellis H. M. Yu D. G. DiTizio T. Court D. L. (2001). High efficiency mutagenesis, repair, and engineering of chromosomal DNA using single-stranded oligonucleotides. *Proc. Natl. Acad. Sci. U.S.A.* 98 6742–6746. 10.1073/pnas.121164898 11381128 PMC34423

[B18] Englaender J. A. Jones J. A. Cress B. F. Kuhlman T. E. Linhardt R. J. Koffas M. A. G. (2017). Effect of genomic integration location on heterologous protein expression and metabolic engineering in *E. coli*. *ACS Synth. Biol.* 6 710–720. 10.1021/acssynbio.6b00350 28055177

[B19] Farhi M. Marhevka E. Masci T. Marcos E. Eyal Y. Ovadis M. (2011). Harnessing yeast subcellular compartments for the production of plant terpenoids. *Metab. Eng.* 13 474–481. 10.1016/j.ymben.2011.05.001 21601648

[B20] Galdieri L. Zhang T. Rogerson D. Lleshi R. Vancura A. (2014). Protein acetylation and acetyl coenzyme a metabolism in budding yeast. *Eukaryot. Cell* 13 1472–1483. 10.1128/ec.00189-14 25326522 PMC4248685

[B21] Gilbert L. A. Larson M. H. Morsut L. Liu Z. Brar G. A. Torres S. E. (2013). CRISPR-mediated modular RNA-guided regulation of transcription in Eukaryotes. *Cell* 154 442–451. 10.1016/j.cell.2013.06.044 23849981 PMC3770145

[B22] Gou M. Ran X. Martin D. W. Liu C.-J. (2018). The scaffold proteins of lignin biosynthetic cytochrome P450 enzymes. *Nat. Plants* 4 299–310. 10.1038/s41477-018-0142-9 29725099

[B23] Gowers G. O. F. Chee S. M. Bell D. Suckling L. Kern M. Tew D. (2020). Improved betulinic acid biosynthesis using synthetic yeast chromosome recombination and semi-automated rapid LC-MS screening. *Nat. Commun.* 11:868. 10.1038/s41467-020-14708-z 32054834 PMC7018806

[B24] Greisman H. A. Pabo C. O. (1997). A general strategy for selecting high-affinity zinc finger proteins for diverse DNA target sites. *Science* 275 657–661. 10.1126/science.275.5300.657 9005850

[B25] Guo X.-J. Xiao W.-H. Wang Y. Yao M.-D. Zeng B.-X. Liu H. (2018). Metabolic engineering of *Saccharomyces cerevisiae* for 7-dehydrocholesterol overproduction. *Biotechnol. Biofuels* 11:192. 10.1186/s13068-018-1194-9 30026807 PMC6047132

[B26] Henriques de Jesus M. P. R. Zygadlo Nielsen A. Busck Mellor S. Matthes A. Burow M. Robinson C. (2017). Tat proteins as novel thylakoid membrane anchors organize a biosynthetic pathway in chloroplasts and increase product yield 5-fold. *Metab. Eng.* 44 108–116. 10.1016/j.ymben.2017.09.014 28962875

[B27] Hochrein L. Mitchell L. A. Schulz K. Messerschmidt K. Mueller-Roeber B. (2018). L-SCRaMbLE as a tool for light-controlled Cre-mediated recombination in yeast. *Nat. Commun.* 9:1931. 10.1038/s41467-017-02208-6 29789561 PMC5964156

[B28] Horn A. H. C. Sticht H. (2015). Synthetic protein scaffolds based on peptide motifs and cognate adaptor domains for improving metabolic productivity. *Front. Bioeng. Biotechnol.* 3:191. 10.3389/fbioe.2015.00191 26636078 PMC4655305

[B29] Hou S. Qin Q. Dai J. (2018). Wicket: a versatile tool for the integration and optimization of exogenous pathways in *Saccharomyces cerevisiae*. *ACS Synth. Biol.* 7 782–788. 10.1021/acssynbio.7b00391 29474063

[B30] Huttanus H. M. Feng X. (2017). Compartmentalized metabolic engineering for biochemical and biofuel production. *Biotechnol. J.* 12:1700052. 10.1002/biot.201700052 28464535

[B31] Jakounas T. Sonde I. Herrgard M. Harrison S. J. Kristensen M. Pedersen L. E. (2015). Multiplex metabolic pathway engineering using CRISPR/Cas9 in *Saccharomyces cerevisiae*. *Metab. Eng.* 28 213–222. 10.1016/j.ymben.2015.01.008 25638686

[B32] Jensen E. D. Ferreira R. Jakociunas T. Arsovska D. Zhang J. Ding L. (2017). Transcriptional reprogramming in yeast using dCas9 and combinatorial gRNA strategies. *Microb. Cell Fact.* 16:46. 10.1186/s12934-017-0664-2 28298224 PMC5353793

[B33] Jia B. Wu Y. Li B. Z. Mitchell L. A. Liu H. Pan S. (2018). Precise control of SCRaMbLE in synthetic haploid and diploid yeast. *Nat. Commun.* 9:1933. 10.1038/s41467-018-03084-4 29789567 PMC5964104

[B34] Jin J. Ma Y. Liu D. (2018). SCRaMbLE drive application of synthetic yeast genome. *Front. Chem. Sci. Eng.* 12:832–834. 10.1007/s11705-018-1749-0

[B35] Kang S.-Y. Heo K. T. Hong Y.-S. (2018). Optimization of artificial curcumin biosynthesis in *E. coli* by randomized 5’-UTR sequences to control the multienzyme pathway. *ACS Synth. Biol.* 7 2054–2062. 10.1021/acssynbio.8b00198 30160937

[B36] Kannan K. Gibson D. G. (2017). Yeast genome, by design. *Science* 355 1024–1025. 10.1126/science.aam9739 28280169

[B37] Keasling J. D. (2012). Synthetic biology and the development of tools for metabolic engineering. *Metab. Eng* 14 189–195. 10.1016/j.ymben.2012.01.004 22314049

[B38] Kim S. K. Han G. H. Seong W. Kim H. Kim S. W. Lee D. H. (2016). CRISPR interference-guided balancing of a biosynthetic mevalonate pathway increases terpenoid production. *Metab. Eng.* 38 228–240. 10.1016/j.ymben.2016.08.006 27569599

[B39] Kovacs W. J. Olivier L. M. Krisans S. K. (2002). Central role of peroxisomes in isoprenoid biosynthesis. *Prog. Lipid Res.* 41 369–391. 10.1016/S0163-7827(02)00002-4 12121718

[B40] Li Y. Lin Z. Huang C. Zhang Y. Wang Z. Tang Y. J. (2015). Metabolic engineering of *Escherichia coli* using CRISPR-Cas9 meditated genome editing. *Metab. Eng.* 31 13–21. 10.1016/j.ymben.2015.06.006 26141150

[B41] Li Y. Wu Y. Ma L. Guo Z. Xiao W. Yuan Y. (2019). Loss of heterozygosity by SCRaMbLEing. *Sci. China Life Sci.* 62 381–393. 10.1007/s11427-019-9504-5 30900161

[B42] Li Z. H. Meng H. Ma B. Tao X. Liu M. Wang F. Q. (2020). Immediate, multiplexed and sequential genome engineering facilitated by CRISPR/Cas9 in *Saccharomyces cerevisiae*. *J. Ind. Microbiol. Biotechnol.* 47 83–96. 10.1007/s10295-019-02251-w 31768773

[B43] Lian J. HamediRad M. Hu S. Zhao H. (2017). Combinatorial metabolic engineering using an orthogonal tri-functional CRISPR system. *Nat. Commun.* 8:1688. 10.1038/s41467-017-01695-x 29167442 PMC5700065

[B44] Lin J. L. Zhu J. Wheeldon I. (2017). Synthetic protein scaffolds for biosynthetic pathway co-localization on lipid droplet membranes. *ACS Synth. Biol.* 6 1534–1544. 10.1021/acssynbio.7b00041 28497697

[B45] Liu H. Fan J. Wang C. Li C. Zhou X. (2019). Enhanced β-amyrin synthesis in *Saccharomyces cerevisiae* by coupling an optimal acetyl-CoA supply pathway. *J. Agric. Food Chem.* 67 3723–3732. 10.1021/acs.jafc.9b00653 30808164

[B46] Liu W. Luo Z. Wang Y. Pham N. T. Tuck L. Pérez-Pi I. (2018). Rapid pathway prototyping and engineering using in vitro and *in vivo* synthetic genome SCRaMbLE-in methods. *Nat. Commun.* 9:1936. 10.1038/s41467-018-04254-0 29789543 PMC5964202

[B47] Liu X. Ding W. Jiang H. (2017). Engineering microbial cell factories for the production of plant natural products: from design principles to industrial-scale production. *Microb. Cell Fact.* 16:125. 10.1186/s12934-017-0732-7 28724386 PMC5518134

[B48] Lü B. Yang X. Feng X. Li C. (2016). Enhanced production of glycyrrhetic acid 3-O-mono-β-d-glucuronide by fed-batch fermentation using pH and dissolved oxygen as feedback parameters. *Chinese J. Chem. Eng.* 24 506–512. 10.1016/j.cjche.2015.12.003

[B49] Lv X. Wang F. Zhou P. Ye L. Xie W. Xu H. (2016). Dual regulation of cytoplasmic and mitochondrial acetyl-CoA utilization for improved isoprene production in *Saccharomyces cerevisiae*. *Nat. Commun.* 7:12851. 10.1038/ncomms12851 27650330 PMC5036000

[B50] Ma L. Li Y. Chen X. Ding M. Wu Y. Yuan Y.-J. (2019). SCRaMbLE generates evolved yeasts with increased alkali tolerance. *Microb. Cell Fact.* 18:52. 10.1186/s12934-019-1102-4 30857530 PMC6410612

[B51] Marienhagen J. Bott M. (2012). Metabolic engineering of microorganisms for the synthesis of plant natural products. *J. Biotechnol.* 163 166–178. 10.1016/j.jbiotec.2012.06.001 22687248

[B52] Mikkelsen M. D. Buron L. D. Salomonsen B. Olsen C. E. Hansen B. G. Mortensen U. H. (2012). Microbial production of indolylglucosinolate through engineering of a multi-gene pathway in a versatile yeast expression platform. *Metab. Eng.* 14 104–111. 10.1016/j.ymben.2012.01.006 22326477

[B53] Morgado G. Gerngross D. Roberts T. M. Panke S. (2018). “Synthetic biology for cell-free biosynthesis: fundamentals of designing novel in vitro multi- enzyme reaction networks,” in *Synthetic Biology - Metabolic Engineering*, eds Zhao H. Zeng A. P. (Cham: Springer), 117–146. 10.1007/10_2016_13 27757475

[B54] Moscou M. J. Bogdanove A. J. (2009). A simple cipher governs DNA recognition by TAL effectors. *Science* 326:1501. 10.1126/science.1178817 19933106

[B55] Ng C. Y. Farasat I. Maranas C. D. Salis H. M. (2015). Rational design of a synthetic Entner–Doudoroff pathway for improved and controllable NADPH regeneration. *Metab. Eng.* 29 86–96. 10.1016/j.ymben.2015.03.001 25769287

[B56] Ni J. Zhang G. Qin L. Li J. Li C. (2019). Simultaneously down-regulation of multiplex branch pathways using CRISPRi and fermentation optimization for enhancing β-amyrin production in *Saccharomyces cerevisiae*. *Synth. Syst. Biotechnol.* 4 79–85. 10.1016/j.synbio.2019.02.002 30949594 PMC6428687

[B57] Ottaviani A. Gilson E. Magdinier F. (2008). Telomeric position effect: from the yeast paradigm to human pathologies? *Biochimie* 90 93–107. 10.1016/j.biochi.2007.07.022 17868970

[B58] Park S. Y. Yang D. Ha S. H. Lee S. Y. (2018). Metabolic engineering of microorganisms for the production of natural compounds. *Adv. Biosyst.* 2:1700190 10.1002/adbi.201700190

[B59] Qi D.-D. Jin J. Liu D. Jia B. Yuan Y.-J. (2020). In vitro and *in vivo* recombination of heterologous modules for improving biosynthesis of astaxanthin in yeast. *Microb. Cell Fact.* 19:103. 10.1186/s12934-020-01356-7 32398013 PMC7216642

[B60] Qu J. Cao S. Wei Q. Zhang H. Wang R. Kang W. (2019). Synthetic multienzyme complexes, catalytic nanomachineries for cascade biosynthesis *in vivo*. *ACS Nano* 13 9895–9906. 10.1021/acsnano.9b03631 31356751

[B61] Quintin M. Ma N. J. Ahmed S. Bhatia S. Lewis A. Isaacs F. J. (2016). Merlin: computer-aided oligonucleotide design for large scale genome engineering with MAGE. *ACS Synth. Biol.* 5 452–458. 10.1021/acssynbio.5b00219 27054880

[B62] Raman S. Rogers J. K. Taylor N. D. Church G. M. (2014). Evolution-guided optimization of biosynthetic pathways. *Proc. Natl. Acad. Sci. U.S.A.* 111 17803–17808. 10.1073/pnas.1409523111 25453111 PMC4273373

[B63] Ronda C. Maury J. Jakociunas T. Jacobsen S. A. B. Germann S. M. Harrison S. J. (2015). CrEdit: CRISPR mediated multi-loci gene integration in *Saccharomyces cerevisiae*. *Microb. Cell Fact.* 14:97. 10.1186/s12934-015-0288-3 26148499 PMC4492099

[B64] Ryu Y. S. Biswas R. K. Shin K. Parisutham V. Kim S. M. Lee S. K. (2014). A simple and effective method for construction of *Escherichia coli* strains proficient for genome engineering. *PLoS One* 9:e94266. 10.1371/journal.pone.0094266 24747264 PMC3991648

[B65] Sachdeva G. Garg A. Godding D. Way J. C. Silver P. A. (2014). *In vivo* co-localization of enzymes on RNA scaffolds increases metabolic production in a geometrically dependent manner. *Nucleic Acids Res.* 42 9493–9503. 10.1093/nar/gku617 25034694 PMC4132732

[B66] Schwartz C. Shabbir-Hussain M. Frogue K. Blenner M. Wheeldon I. (2017). Standardized markerless gene integration for pathway engineering in *Yarrowia lipolytica*. *ACS Synth. Biol.* 6 402–409. 10.1021/acssynbio.6b00285 27989123

[B67] Si T. Chao R. Min Y. Wu Y. Ren W. Zhao H. (2017). Automated multiplex genome-scale engineering in yeast. *Nat. Commun.* 8:15187. 10.1038/ncomms15187 28469255 PMC5418614

[B68] Siemon T. Wang Z. Bian G. Seitz T. Ye Z. Lu Y. (2020). Semisynthesis of plant-derived Englerin A enabled by microbe engineering of guaia-6,10(14)-diene as building block. *J. Am. Chem. Soc.* 142 2760–2765. 10.1021/jacs.9b12940 31999448

[B69] Standage-Beier K. Wang X. (2017). Genome reprogramming for synthetic biology. *Front. Chem. Sci. Eng.* 11 37–45. 10.1007/s11705-017-1618-2

[B70] Sun W. Qin L. Xue H. Yu Y. Ma Y. Wang Y. (2019). Novel trends for producing plant triterpenoids in yeast. *Crit. Rev. Biotechnol.* 39 618–632. 10.1080/07388551.2019.1608503 31068012

[B71] Sun W. Xue H. Liu H. Lv B. Yu Y. Wang Y. (2020). Controlling chemo- and regioselectivity of a plant P450 in yeast cell toward rare licorice triterpenoid biosynthesis. *ACS Catal.* 10 4253–4260. 10.1021/acscatal.0c00128

[B72] Thompson A. Gasson M. J. (2001). Location effects of a reporter gene on expression levels and on native protein synthesis in *Lactococcus lactis* and *Saccharomyces cerevisiae*. *Appl. Environ. Microbiol.* 67 3434–3439. 10.1128/aem.67.8.3434-3439.2001 11472915 PMC93039

[B73] Wang H. H. Isaacs F. J. Carr P. A. Sun Z. Z. Xu G. Forest C. R. (2009). Programming cells by multiplex genome engineering and accelerated evolution. *Nature* 460 894–898. 10.1038/nature08187 19633652 PMC4590770

[B74] Wang H. H. Kim H. Cong L. Jeong J. Bang D. Church G. M. (2012). Genome-scale promoter engineering by coselection MAGE. *Nat. Methods* 9 591–596. 10.1038/nmeth.1971 22484848 PMC3428217

[B75] Wang J. Jia B. Xie Z. Li Y. Yuan Y. (2018). Improving prodeoxyviolacein production via multiplex SCRaMbLE iterative cycles. *Front. Chem. Sci. Eng.* 12 806–814. 10.1007/s11705-018-1739-2

[B76] Wang L. Deng A. Zhang Y. Liu S. Liang Y. Bai H. (2018). Efficient CRISPR-Cas9 mediated multiplex genome editing in yeasts. *Biotechnol. Biofuels* 11:277. 10.1186/s13068-018-1271-0 30337956 PMC6180501

[B77] Wang Y. Yu O. (2012). Synthetic scaffolds increased resveratrol biosynthesis in engineered yeast cells. *J. Biotechnol.* 157 258–260. 10.1016/j.jbiotec.2011.11.003 22100267

[B78] Wu J. Du G. Chen J. Zhou J. (2015). Enhancing flavonoid production by systematically tuning the central metabolic pathways based on a CRISPR interference system in *Escherichia coli*. *Sci. Rep.* 5:13477. 10.1038/srep13477 26323217 PMC4555050

[B79] Wu J. Zhang X. Zhu Y. Tan Q. He J. Dong M. (2017a). Rational modular design of metabolic network for efficient production of plant polyphenol pinosylvin. *Sci. Rep.* 7:1459. 10.1038/s41598-017-01700-9 28469159 PMC5431097

[B80] Wu J. Zhou P. Zhang X. Dong M. (2017b). Efficient de novo synthesis of resveratrol by metabolically engineered *Escherichia coli*. *J. Ind. Microbiol. Biotehnol.* 44 1083–1095. 10.1007/s10295-017-1937-9 28324236

[B81] Wu X.-L. Li B.-Z. Zhang W.-Z. Song K. Qi H. Dai J. B. (2017). Genome-wide landscape of position effects on heterogeneous gene expression in *Saccharomyces cerevisiae*. *Biotechnol. Biofuels* 10:189. 10.1186/s13068-017-0872-3 28729884 PMC5516366

[B82] Xie S. S. Qiu X. Y. Zhu L. Y. Zhu C. S. Liu C. Y. Wu X. M. (2019). Assembly of TALE-based DNA scaffold for the enhancement of exogenous multi-enzymatic pathway. *J. Biotechnol.* 296 69–74. 10.1016/j.jbiotec.2019.03.008 30885657

[B83] Xie W. Liu M. Lv X. Lu W. Gu J. Yu H. (2014). Construction of a controllable beta-carotene biosynthetic pathway by decentralized assembly strategy in *Saccharomyces cerevisiae*. *Biotechnol. Bioeng.* 111 125–133. 10.1002/bit.25002 23860829

[B84] Xu X. Tian L. Tang S. Xie C. Xu J. Jiang L. (2020). Design and tailoring of an artificial DNA scaffolding system for efficient lycopene synthesis using zinc-finger-guided assembly. *J. Ind. Microbiol. Biotechnol.* 47 209–222. 10.1007/s10295-019-02255-6 31853777

[B85] Yan Y. J. Kohli A. Koffas M. A. G. (2005). Biosynthesis of natural flavanones in *Saccharomyces cerevisiae*. *Appl. Environ. Microbiol.* 71 5610–5613. 10.1128/aem.71.9.5610-5613.2005 16151160 PMC1214632

[B86] Ye L. Lv X. Yu H. (2017). Assembly of biosynthetic pathways in *Saccharomyces cerevisiae* using a marker recyclable integrative plasmid toolbox. *Front. Chem. Sci. Eng.* 11 126–132. 10.1007/s11705-016-1597-8

[B87] Yee D. A. DeNicola A. B. Billingsley J. M. Creso J. G. Subrahmanyam V. Tang Y. (2019). Engineered mitochondrial production of monoterpenes in *Saccharomyces cerevisiae*. *Metab. Eng.* 55 76–84. 10.1016/j.ymben.2019.06.004 31226348 PMC6717016

[B88] Yu Y. Chang P. Yu H. Ren H. Hong D. Li Z. (2018). Productive amyrin synthases for efficient alpha-amyrin synthesis in engineered *Saccharomyces cerevisiae*. *ACS Synth. Biol.* 7 2391–2402. 10.1021/acssynbio.8b00176 30216049

[B89] Yuan J. Ching C. B. (2016). Mitochondrial acetyl-CoA utilization pathway for terpenoid productions. *Metab. Eng.* 38 303–309. 10.1016/j.ymben.2016.07.008 27471067

[B90] Zalatan J. G. Lee M. E. Almeida R. Gilbert L. A. Whitehead E. H. La Russa M. (2015). Engineering complex synthetic transcriptional programs with CRISPR RNA scaffolds. *Cell* 160 339–350. 10.1016/j.cell.2014.11.052 25533786 PMC4297522

[B91] Zhang C. Li M. Zhao G.-R. Lu W. (2020). Harnessing yeast peroxisomes and cytosol acetyl-coA for sesquiterpene α-humulene production. *J. Agric. Food Chem.* 68 1382–1389. 10.1021/acs.jafc.9b07290 31944688

[B92] Zhang G. Cao Q. Liu J. Liu B. Li J. Li C. (2015). Refactoring β-amyrin synthesis in *Saccharomyces cerevisiae*. *AIChE J.* 61 3172–3179. 10.1002/aic.14950

[B93] Zhang L. Gao Y. Liu X. Guo F. Ma C. Liang J. (2019). Mining of sucrose synthases from Glycyrrhiza uralensis and their application in the construction of an efficient UDP-recycling system. *J. Agric. Food Chem.* 67 11694–11702. 10.1021/acs.jafc.9b05178 31558015

[B94] Zhang L. Zhang H. Liu Y. Zhou J. Shen W. Liu L. (2020). A CRISPR–Cas9 system for multiple genome editing and pathway assembly in *Candida tropicalis*. *Biotechnol. Bioeng.* 117 531–542. 10.1002/bit.27207 31654413

[B95] Zhang X. K. Wang D. N. Chen J. Liu Z. J. Wei L. J. Hua Q. (2020). Metabolic engineering of β-carotene biosynthesis in *Yarrowia lipolytica*. *Biotechnol. Lett.* 42 945–956. 10.1007/s10529-020-02844-x 32090297

[B96] Zhang Y. Wang J. Cao X. Liu W. Yu H. Ye L. (2020). High-level production of linalool by engineered *Saccharomyces cerevisiae* harboring dual mevalonate pathways in mitochondria and cytoplasm. *Enzyme Microb. Technol.* 134:109462. 10.1016/j.enzmictec.2019.109462 32044019

[B97] Zhang Y. Wang J. Wang Z. Zhang Y. Shi S. Nielsen J. (2019). A gRNA-tRNA array for CRISPR-Cas9 based rapid multiplexed genome editing in *Saccharomyces cerevisiae*. *Nat. Commun.* 10:1053. 10.1038/s41467-019-09005-3 30837474 PMC6400946

[B98] Zhang Y. H. P. (2011). Substrate channeling and enzyme complexes for biotechnological applications. *Biotechnol. Adv.* 29 715–725. 10.1016/j.biotechadv.2011.05.020 21672618

[B99] Zhao S. Jones J. A. Lachance D. M. Bhan N. Khalidi O. Venkataraman S. (2015). Improvement of catechin production in *Escherichia coli* through combinatorial metabolic engineering. *Metab. Eng.* 28 43–53. 10.1016/j.ymben.2014.12.002 25527438

[B100] Zhao Y. Fan J. Wang C. Feng X. Li C. (2018). Enhancing oleanolic acid production in engineered *Saccharomyces cerevisiae*. *Bioresour. Technol.* 257 339–343. 10.1016/j.biortech.2018.02.096 29526355

[B101] Zhao Y. Lv B. Feng X. Li C. (2017). Perspective on biotransformation and de novo biosynthesis of licorice constituents. *J. Agric. Food Chem.* 65 11147–11156. 10.1021/acs.jafc.7b04470 29179542

[B102] Zhao Y.-J. Li C. (2018). Biosynthesis of plant triterpenoid saponins in microbial cell factories. *J. Agric. Food Chem.* 66 12155–12165. 10.1021/acs.jafc.8b04657 30387353

[B103] Zhou P. Xie W. Yao Z. Zhu Y. Ye L. Yu H. (2018). Development of a temperature-responsive yeast cell factory using engineered Gal4 as a protein switch. *Biotechnol. Bioeng.* 115 1321–1330. 10.1002/bit.26544 29315481

[B104] Zhu L. Y. Qiu X. Y. Zhu L. Y. Wu X. M. Zhang Y. Zhu Q. H. (2016). Spatial organization of heterologous metabolic system *in vivo* based on TALE. *Sci. Rep.* 6:26065. 10.1038/srep26065 27184291 PMC4869064

[B105] Zhu M. Wang C. Sun W. Zhou A. Wang Y. Zhang G. (2018). Boosting 11-oxo-β-amyrin and glycyrrhetinic acid synthesis in *Saccharomyces cerevisiae* via pairing novel oxidation and reduction system from legume plants. *Metab. Eng.* 45 43–50. 10.1016/j.ymben.2017.11.009 29196123

