# Supplementary material for: Microbial Cell Factory for Efficiently Synthesizing Plant Natural Products via Optimizing the Location and Adaptation of Pathway on Genome Scale
Source: Front Bioeng Biotechnol. 2020 Aug 14;8:969. doi: 10.3389/fbioe.2020.00969 (PMC7457125; doi:10.3389/fbioe.2020.00969)
Supplement: Supplementary file 1 [file Table_1.pdf]

Supplementary Material

1 Supplementary Table

Table S1 Comparison of three types of synthetic scaffolds

Synthetic scaffold	Strengths	Weaknesses
DNA scaffold	High extensibility, sequence-independent stability	Lack of rigidity
RNA scaffold	Predictability, flexibility, few undesirable crosstalk	Degradability, less RNA binding domains with high binding affinity
Protein scaffold	More positions for enzyme colocalization	The spatial arrangement of the target protein is affected by the structure of scaffold protein
